# Ubiquilin-2 regulates pathological alpha-synuclein

**DOI:** 10.1038/s41598-022-26899-0

**Published:** 2023-01-06

**Authors:** Stephanie S. Sandoval-Pistorius, Julia E. Gerson, Nyjerus Liggans, Jaimie H. Ryou, Kulin Oak, Xingli Li, Keyshla Y. Negron-Rios, Svetlana Fischer, Henry Barsh, Emily V. Crowley, Mary E. Skinner, Lisa M. Sharkey, Sami J. Barmada, Henry L. Paulson

**Affiliations:** 1 Department of Neurology, University of Michigan, Ann Arbor, MI 48109-2200 USA; 2 Neuroscience Graduate Program, University of Michigan, Ann Arbor, MI 48109 USA

**Keywords:** Parkinson's disease, Neurodegeneration, Molecular neuroscience

## Abstract

The key protein implicated in Parkinson’s disease and other synucleinopathies is α-synuclein, and a post-translationally modified form of the protein, phosphorylated at serine 129 (pS129), is a principal component in Lewy bodies, a pathological hallmark of PD. While altered proteostasis has been implicated in the etiology of Parkinson’s disease, we still have a limited understanding of how α-synuclein is regulated in the nervous system. The protein quality control protein Ubiquilin-2 (UBQLN2) is known to accumulate in synucleinopathies, but whether it directly regulates α-synuclein is unknown. Using cellular and mouse models, we find that UBQLN2 decreases levels of α-synuclein, including the pS129 phosphorylated isoform. Pharmacological inhibition of the proteasome revealed that, while α-synuclein may be cleared by parallel and redundant quality control pathways, UBQLN2 preferentially targets pS129 for proteasomal degradation. Moreover, in brain tissue from human PD and transgenic mice expressing pathogenic α-synuclein (A53T), native UBQLN2 becomes more insoluble. Collectively, our studies support a role for UBQLN2 in directly regulating pathological forms of α-synuclein and indicate that UBQLN2 dysregulation in disease may contribute to α-synuclein-mediated toxicity.

## Introduction

Like many neurodegenerative diseases, Parkinson’s disease (PD) and Lewy Body Dementia (DLB) are characterized by the accumulation and misfolding of disease-specific proteins^1^. A key pathological feature of PD and DLB is the presence of α-synuclein (α-syn)-rich Lewy bodies (LBs) and Lewy neurites (LNs) in neurons of affected brain regions^2,3^. Several factors contribute to the accumulation of aggregated α-syn in synucleinopathies, including post-translational modifications (such as phosphorylation at serine 129), increased production (duplication or triplication of the *SNCA* gene), missense mutations (e.g., A30P and A53T)^4,5^ and altered degradation of α-syn protein^6^. Perturbations in proteostasis have been implicated in the pathogenesis of synucleinopathies^7^. Evidence supports regulation of α-syn by multiple quality control systems, including proteasomal and lysosomal pathways^6,8,9^. The specific aggregation state of α-syn may also influence which pathway(s) it is handled by.

Ubiquilin-2 (UBQLN2), a protein quality control protein, has been linked to PD and DLB due to its colocalization with accumulated α-syn in PD and DLB post-mortem tissue^10^. UBQLN2 is one member of the family of ubiquilins that function as shuttle proteins for the ubiquitin–proteasome system (UPS)^11^. Ubiquilins are similar in structure, containing N terminus ubiquitin-like (UBL) and C-terminus ubiquitin-associated (UBA) domains that facilitate the shuttling of ubiquitinated substrate proteins to the proteasome for degradation^12,13^. UBQLNs also contain STI1 motifs that may mediate binding to chaperones^14^. Among the UBQLNs, UBQLN2 uniquely contains a proline-rich domain (PXX)^15^. The prevailing view is that UBQLNs function predominantly as UPS shuttle proteins but likely participate in other quality control pathways as well, including autophagy^11,16–18^.

The importance of UBQLNs in neurodegenerative disease was underscored by the discovery that mutations in UBQLN2 directly cause frontotemporal dementia (FTD) and amyotrophic lateral sclerosis (ALS)^15,19,20^. UBQLN2 also forms liquid condensates and can aggregate in vitro^17,21^ and in vivo^22^, with pathogenic mutations increasing UBQLN2 aggregation^22–26^. UBQLN2 also co-localizes with aggregates of disease proteins such as huntingtin in Huntington’s disease, and TDP-43 and dipeptide repeat proteins in ALS/FTD^10,15^. Collectively, these results support the view that UBQLN2 contributes to multiple neurodegenerative diseases. Precisely how, however, is poorly understood.

Outside of the colocalization of UBQLN2 with Lewy bodies in PD and DLB, little is known about the role of UBQLN2 in synucleinopathies. For example, it is unknown whether UBQLN2 sequestration into protein aggregates supports a role for UBQLN2 in regulating α-syn, either normally or in disease states. Here, we use human disease tissue and transgenic mouse and cellular models to show that UBQLN2 regulates a key pathogenic form of α-syn, pS129, by targeting it to the proteasome for degradation.

## UBQLN2 insolubility is increased in PD

**Figure 1:**
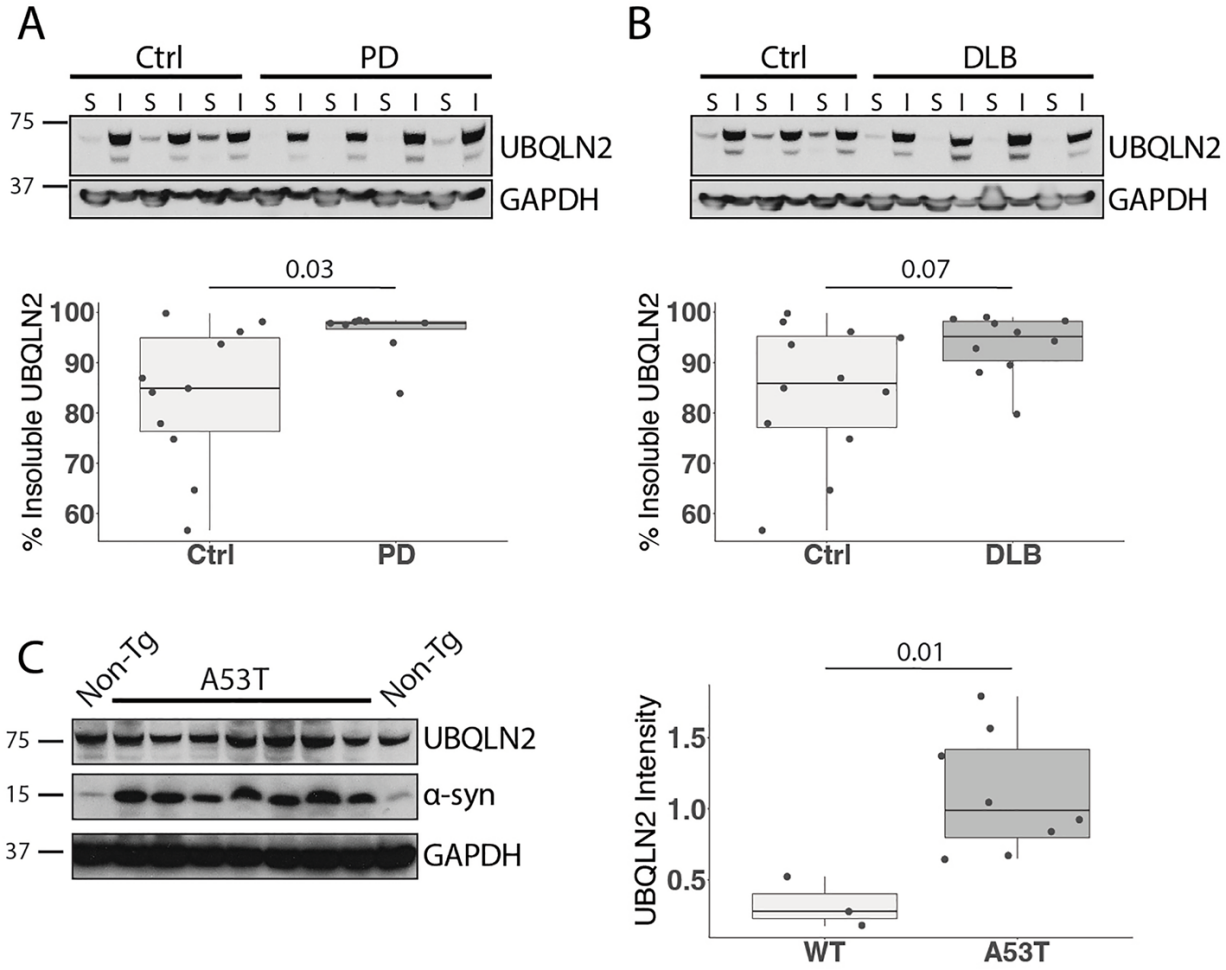
UBQLN2 insolubility is increased in Parkinson’s disease human tissue and mouse models. (**A**,**B**) Western blots of lysates from post-mortem cingulate cortex of PD and DLB patients show an increase in insoluble UBQLN2 in (**A**) PD (n = 8) and (**B**) DLB (n = 10) compared to age-matched controls. *S* PBS soluble, *I* PBS insoluble/sarkosyl soluble. (**C**) Representative western blot of whole brain lysate from mice overexpressing human mutant α-syn A53T solubilized in sarkosyl (n = 8) shows elevated levels of insoluble UBQLN2 compared to non-transgenic controls (n = 3). Western blots were cropped to focus on protein(s) of interest. Uncropped Western blots are shown in Supplemental Figure S1.

Previous studies established that UBQLN2 accumulates in proteinaceous aggregates in several neurodegenerative diseases^10,15^. To determine whether UBQLN2 solubility is altered in synucleinopathies, we assessed soluble and insoluble levels of UBQLN2 in protein lysates from PD and DLB patient cingulate cortex. Compared to age-matched controls, PD patient brain lysates exhibited increased insoluble UBQLN2 (p = 0.03; Fig. 1A) and DLB brain lysates exhibited a trend towards increased insoluble UBQLN2 (p = 0.07; Fig. 1B). Taken together with previous studies showing that UBQLN2 colocalizes with LBs^10^, this finding suggests that UBQLN2 is sequestered in insoluble material.

To determine whether a rodent model of PD recapitulates this observed increase in insoluble UBQLN2 in human disease, we analyzed levels of UBQLN2 in whole brains from 10-month-old transgenic mice expressing a pathogenic form of α-syn (A53T) implicated in familial PD^27^. Similar to our observations in human disease tissue, insoluble UBQLN2 was increased in brains from A53T mice compared to non-transgenic (Non-Tg) controls (p = 0.01; Fig. 1C). This result suggests not only that the A53T transgenic mouse is a relevant model for the study of UBQLN2 in synucleinopathies, but also that UBQLN2 solubility is affected by mutant α-syn.

## UBQLN2, but not UBQLN1, preferentially regulates pS129 α-syn

**Figure 2:**
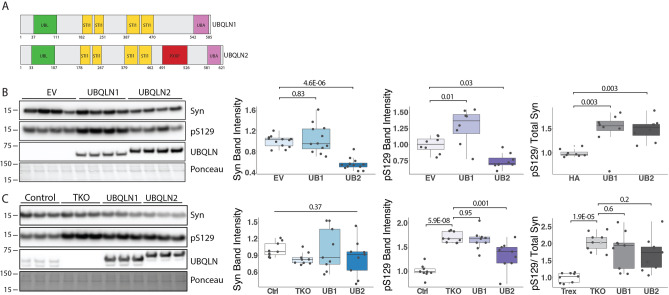
UBQLN KO leads to pS129 accumulation while UBQLN2 overexpression decreases levels of pS129 in vitro. (**A**) Diagram of UBQLN2 and UBQLN1 protein structure. (**B**) Representative western blot of UBQLN 1,2, 4 Total Knock out (TKO) cells transfected to express WT α-syn alone or together with UBQLN1 (UB1) or UBQLN2 (UB2). Total α-syn and pS129 levels are decreased by co-expression of UBQLN2 but not UBQLN1. The ratio of pS129/total Syn is increased in both UBQLN1 and UBQLN2 co-expression (n = 8). (**C**) Representative western blot of TKO, UBQLN1 rescue, UBQLN2 rescue, and control cells were transfected with α-syn showing elevated pS129 levels in TKO cells compared to control cells, which is partially reversed by inducible co-expression of UBQLN2 but not UBQLN1. The ratio of pS129/total Syn is significantly increased in TKO compared to control and not reversed by UBQLN1 or UBQLN2 rescue (n = 8). Western blots were cropped to focus on protein(s) of interest. Uncropped Western blots are shown in Supplemental Figure S2.

UBQLN2’s function as a shuttle protein in protein quality control pathways and its colocalization with various disease protein aggregates^10,15^ suggest that it may regulate misfolded disease proteins such as α-syn by altering protein levels. LBs are enriched for pS129^28,29^, and based on studies showing that UBQLN2 colocalizes with LBs in disease^10^, we hypothesized that UBQLN2 may regulate α-syn, particularly levels of phosphorylated forms such as pS129. Preliminary studies in HEK-293 cells transiently transfected to express UBQLN2 and α-syn supported the view that UBQLN2 decreases levels of α-syn (data not shown). However, HEK-293 cells contain endogenous UBQLNs which can confound analysis. Accordingly, we used UBQLN triple knockout cells (TKO)^30^, which are devoid of UBQLNs 1, 2 and 4 to test the role of UBQLN2, or closely similar UBQLN1, in regulating α-syn, absent any potential compensatory effects of other UBQLNs. We transiently transfected α-syn with plasmids encoding UBQLN1, UBQLN2 or an empty vector (EV) and found that UBQLN2, but not UBQLN1, significantly decreased total α-syn (p ≤ 0.001) and pS129 levels (p = 0.03) compared to EV control (Fig. 2B). UBQLN1 expression led to an accumulation of pS129 levels (p = 0.01; Fig. 2B), but unlike UBQLN2, UBQLN1 has not been broadly implicated in neurodegeneration and does not display altered solubility in PD and DLB^31^. These results point to a preferential effect of UBQLN2 in altering levels of α-syn, including pS129.

UBQLNs function as shuttle proteins for the UPS and form heterodimers that may be important for their function^32^. To assess the role of UBQLN 1 or 2 on α-syn and pS129 protein levels when expressed at closer to endogenous levels and independent of other UBQLNs, we transiently expressed α-syn in TKO UBQLN rescue cells that inducibly express either UBQLN1 or UBQLN2 when treated with doxycycline^30^. These UBQLN rescue lines overexpress UBQLN1 or UBQLN2 to a lesser extent than when transiently transfected, allowing us to assess UBQLN1 and UBQLN2 regulation of α-syn in a more biologically relevant environment. Control cells that express endogenous levels of UBQLN 1, 2 and 4 were also evaluated. In the absence of all UBQLNs, levels of pS129 were significantly increased in TKO cells, and induction of UBQLN2 significantly decreased pS129 levels, compared to TKO cells (p = 0.001; Fig. 2C). In contrast, UBQLN1 induction had no effect on pS129 levels compared to TKO cells (p = 0.95; Fig. 2C), suggesting UBQLN1 may only cause pS129 accumulation when an overabundance of UBQLN1 is expressed. Total α-syn levels were unchanged in TKO and UBQLN 1 or 2 rescue cells compared to control cells (p = 0.37; Fig. 2C). Analysis of the ratio of pS129 to total Syn revealed no significant difference between TKO and UBQLN 1 (p = 0.6; Fig. 2C) or UBQLN 2 (p = 0.2; Fig. 2C). These data further support a role for UBQLN2 in regulating α-syn, including a pathogenic isoform, pS129.

**Figure 3:**
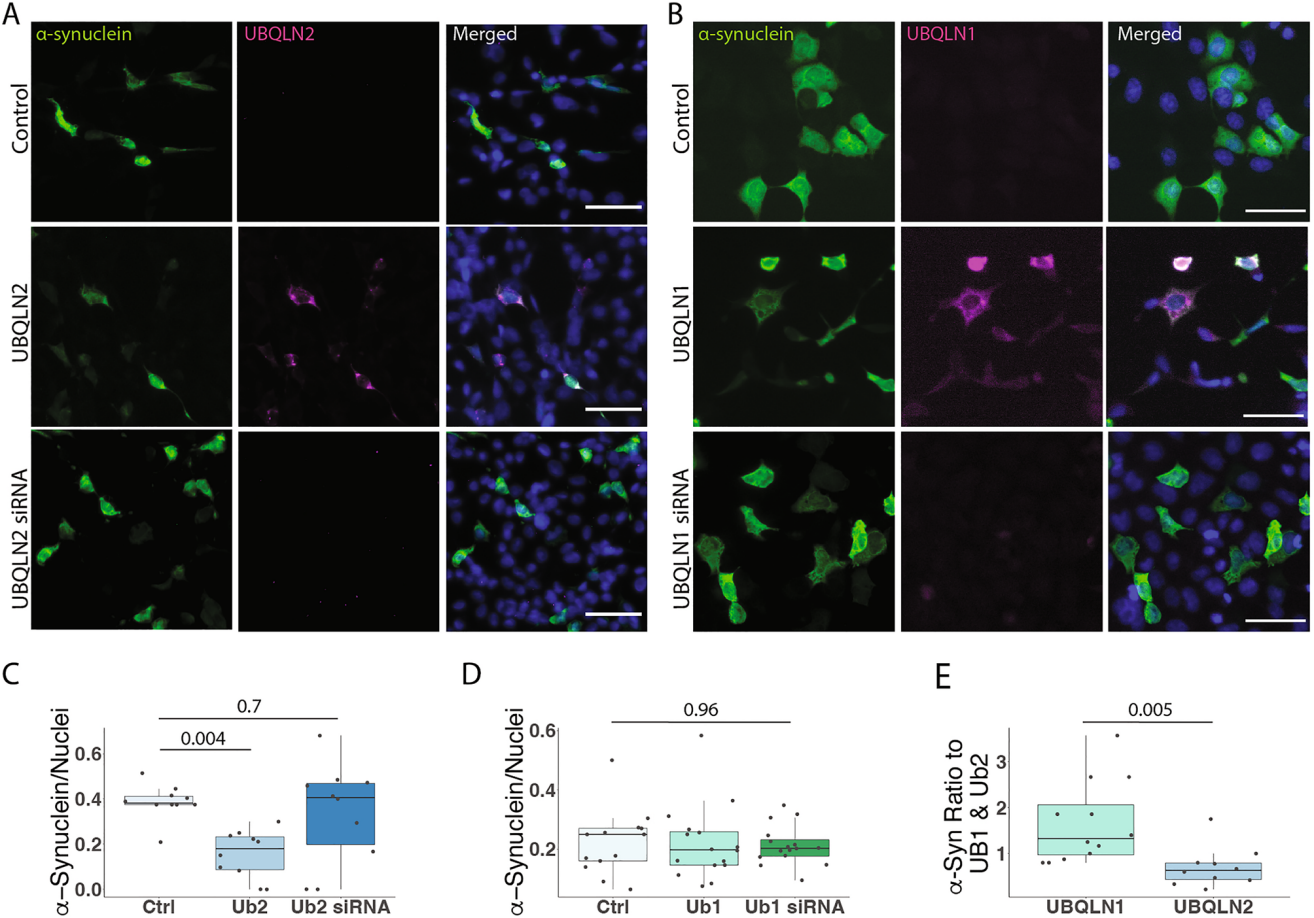
UBQLN2, but not UBQLN1, lowers α-syn levels in HEK-293 cells. (**A**–**D**) Representative immunofluorescence images show that the number of α-syn-containing cells are decreased when co-expressed with UBQLN2 (**A**,**C**; n = 10 field of cells over 3 independent experiments) but not with UBQLN1 (**B**,**D**; n = 10 field of cells over 3 independent experiments). Scale bar 100 μm (**E**) Expressed as a ratio of α-syn positive cells to UBQLN1 and UBQLN2, α-syn is significantly reduced in UBQLN2-expressing cells.

To further explore UBQLN2 regulation of α-syn, we performed immunofluorescence on HEK-293 cells in which we transiently expressed or knocked down UBQLN2 while over-expressing wild-type α-syn. Immunofluorescence analysis supported the finding that UBQLN2 overexpression decreases levels of α-syn (p = 0.004; Fig. 3A,C). However, α-syn levels were unchanged upon UBQLN2 knock-down (p = 0.7; Fig. 3A,C), perhaps due to compensatory effects of other UBQLNs and the presence of remaining UBQLN2 following partial knock-down. In contrast, levels of α-syn were not altered by UBQLN1 overexpression or knock-down (p = 0.96; Fig. 3B,D). A direct comparison of the number of α-syn-positive cells expressed as a ratio to UBQLN2- or UBQLN1-positive cells again showed far fewer α-syn-positive cells with UBQLN2 than with UBQLN1 (p = 0.005; Fig. 3E), further supporting the conclusion that UBQLN2 preferentially regulates α-syn.

## UBQLN2 KO robustly increases the levels of pathogenic pS129 α-syn

**Figure 4:**
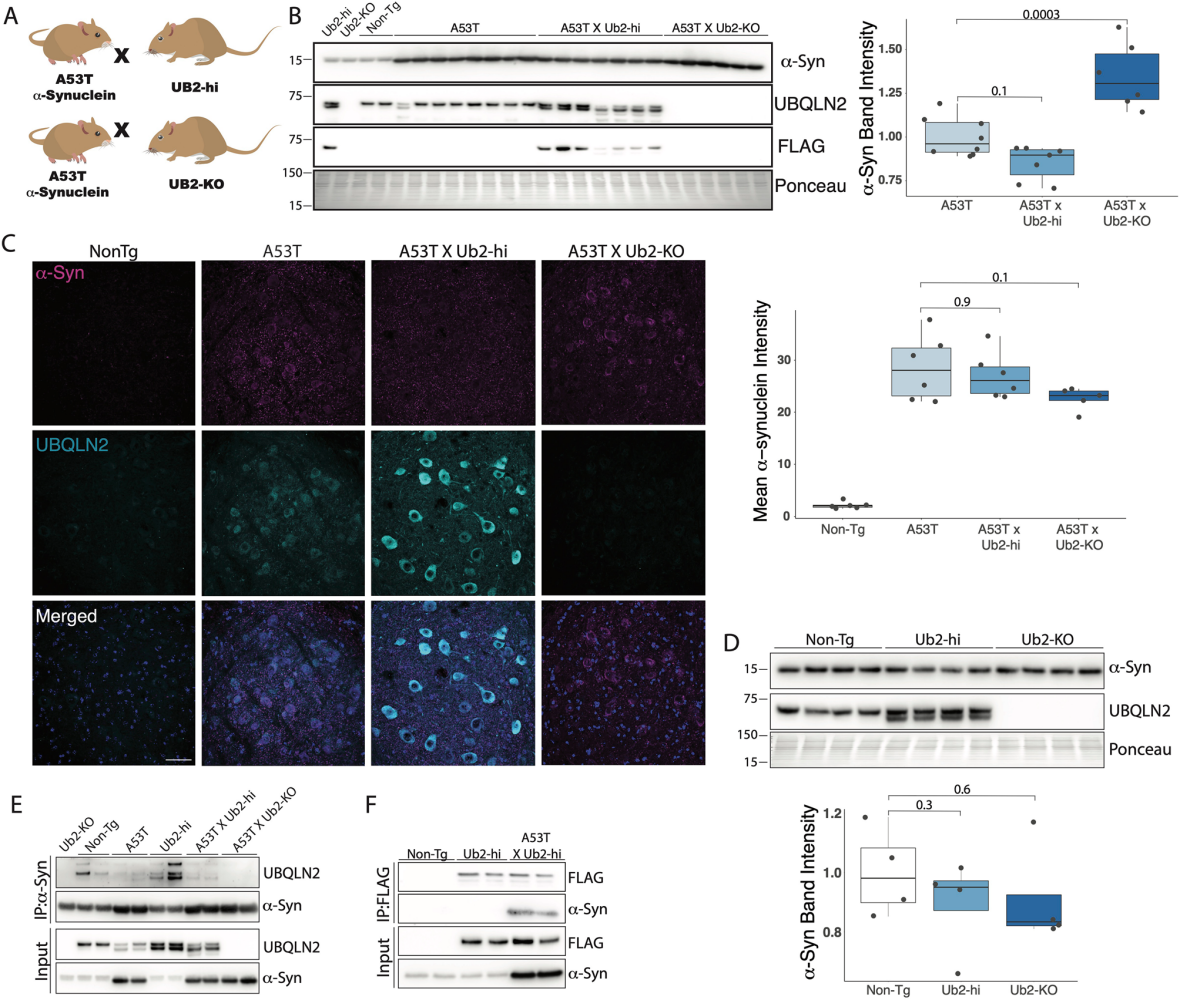
UBQLN2 interacts with and may alter α-syn levels in A53T mice. (**A**) Schematic depicting breeding strategy to obtain A53TxUb2-hi and A53TxUb2-KO mice. (**B**) Western blot of RIPA-soluble brain lysates showing increased levels of total α-syn in A53TxUb2-KO mice (n = 6), while A53TxUb2-hi (n = 7) mice show a trend toward decreased α-syn levels compared to non-transgenic (Non-Tg) controls (n = 8). (**C**) Representative images of total a-syn (magenta) and UBQLN2 (cyan) immunofluorescence in the deep cerebellar nucleus of 12-month-old Non-Tg (n = 6), A53T (n = 6), A53TxUb2-hi (n = 6), and A53TxUb2-KO (n = 5) mice and quantification show no significant difference in total a-syn expression between genotypes. (**D**) Western blot showing total endogenous α-syn levels were unchanged in both Ub2-hi (n = 4) and Ub2-KO mice (n = 4) compared to Non-Tg controls (n = 4). Western blots were cropped to focus on protein(s) of interest. Uncropped Western blots are shown in Supplemental Figure S3). (**E**) Immunoblot showing endogenous and transgenic UBQLN2 is pulled down when α-syn is immunoprecipitated from mouse brain lysate (n = 4). (**F**) Immunoblot showing α-syn coprecipitates with FLAG-tagged UBQLN2 in A53TxUb2-hi mice when FLAG is immunoprecipitated from mouse brain lysate (n = 2).

Our results showing UBQLN2 regulation of α-syn in cell models (Fig. 2) and increasing UBQLN2 insolubility in human and mouse brains harboring α-syn accumulation (Fig. 1), led us to test whether UBQLN2 regulates α-syn in vivo. To assess this, we measured levels of total α-syn in RIPA-soluble brain lysates from transgenic mice expressing pathogenic α-syn (A53T) crossed to either a transgenic mouse line overexpressing UBQLN2 (Ub2-Hi; Fig. 4A) or to the UBQLN2 KO mouse (Ub2-KO; Fig. 4A). Contrary to our in vitro studies (Figs. 1, 2), A53T mice devoid of UBQLN2 (A53TxUb2-KO) displayed an accumulation of total α-syn compared to A53T control mice (p = 0.0003; Fig. 4B), while A53T mice overexpressing UBQLN2 (A53TxUb2-hi) showed a trend toward decreased levels of α-syn compared to A53T (n = 7; p = 0.1). To further assess total α-syn levels in vivo, we performed immunofluorescence to measure levels of α-syn in the deep cerebellar nucleus (DCN), a brain region known to be affected in the A53T mouse model^27^. We did not detect a significant difference in α-syn immunofluorescence between A53T and A53TxUb2-hi (p = 0.9) or A53TxUb2-KO (p = 0.1) mice (Fig. 4C).

To determine if UBQLN2 plays a role in handling α-syn in non-disease states, we measured levels of total endogenous murine α-syn in Ub2-hi and Ub2-KO mice as well as in non-transgenic littermate controls by western blot. We did not detect a significant change in total endogenous α-syn in Ub2-Hi (p = 0.3) or Ub2-KO (p = 0.6) mice compared to controls (Fig. 4D). To determine whether UBQLN2 and α-syn interact, we performed immunoprecipitation assays which captured an interaction between UBQLN2 and α-syn with reciprocal pull downs using either α-syn or UBQLN2 antibodies (Fig. 4E,F). These results suggest that UBQLN2 interacts with α-syn, despite not decreasing levels of total α-syn in vivo.

**Figure 5:**
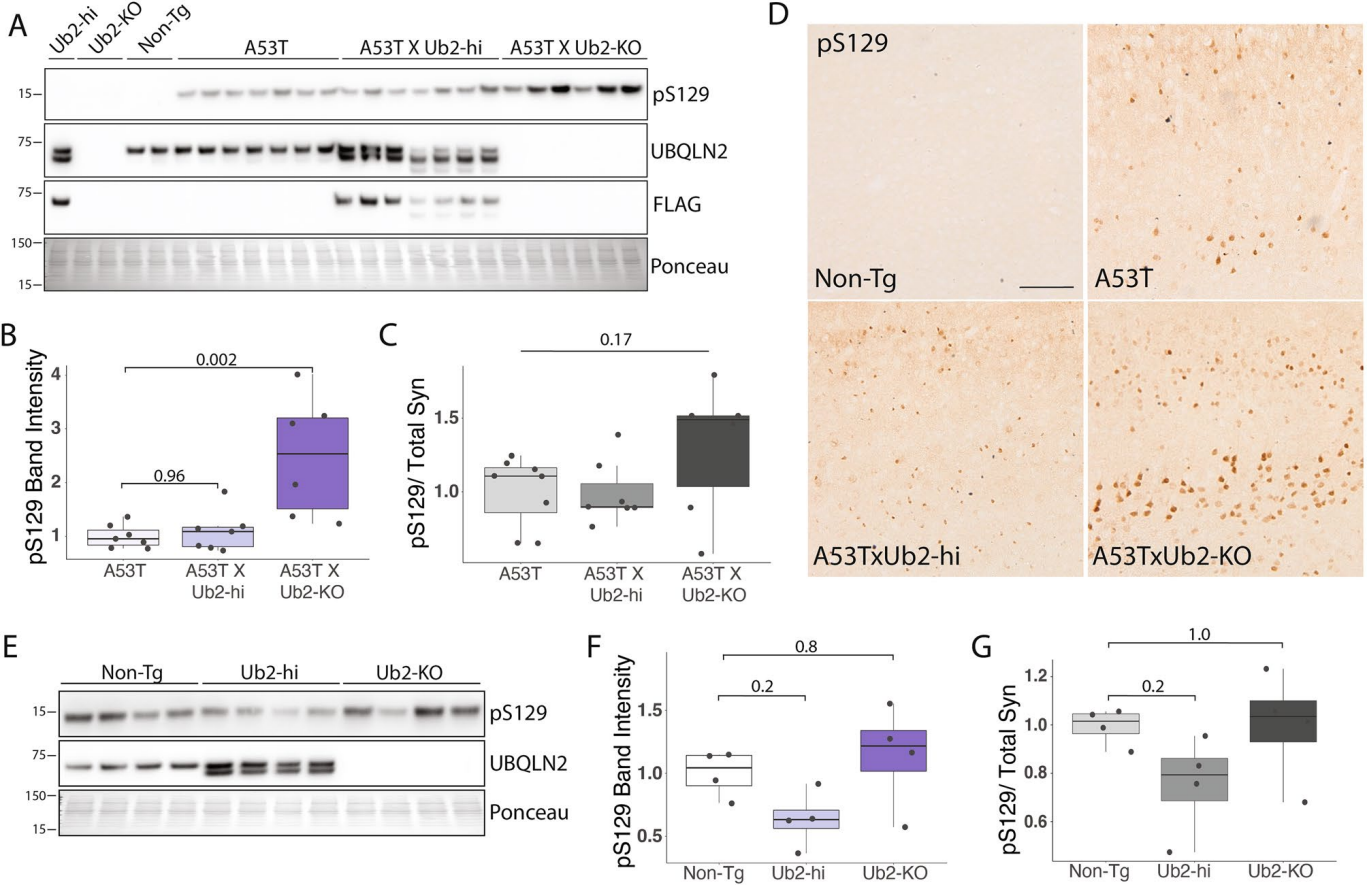
pS129 α-syn accumulates in A53T mice lacking UBQLN2. (**A**) Representative western blot of pS129 in RIPA-soluble brain lysates from the indicated transgenic mouse crosses. (**B**) Quantification from A showing accumulation of pS129 in A53TxUb2-KO mice (n = 6) and unchanged in A53TxUb2-hi mice (n = 7) compared to A53T controls (n = 8). (**C**) Ratio of pS129 to total Syn (see Fig. 4) does not differ significantly between groups (n = 8). (**D**) Representative images of pS129 expression in the cortex of A53T (n = 3), A53TxUb2-hi (n = 3) and A53TxUb2-KO (n = 3) show increased pS129 expression in A53T mice lacking UBQLN2 (12-month-old mice). (**E**) Representative western blots of endogenous pS129 in UBQLN2 transgenic mice. (**F**) Quantification of E showing a trend toward decreased endogenous murine pS129 levels in Ub2-hi mice (n = 4). (**G**) Analysis of the ratio of endogenous pS129 to total endogenous Syn (obtained from Fig. 4) shows no difference between groups (n = 4). Western blots were cropped to focus on protein(s) of interest. Uncropped Western blots are shown in Supplemental Figure S4.

The accumulation and deposition of pS129 α-syn into LBs is a hallmark of synucleinopathies^29^. UBQLN2 can also be found in LBs^10^. To determine whether UBQLN2 regulates levels of pS129 α-syn we measured pS129 levels in A53TxUb2-hi and A53TxUb2-KO mice. Western blot analysis revealed robust accumulation of pS129 in brain lysates of A53TxUb2-KO mice compared to controls (p = 0.002; Fig. 5A,B), while A53TxUb2-hi mice showed no difference in pS129 levels (p = 0.96; Fig. 5A,B). Analysis of the ratio of pS129 to total Syn levels (Fig. 4B) revealed no significant difference in pS129/total Syn between groups (p = 0.17; Fig. 5C). Further assessment of pS129 levels by immunohistochemistry revealed an accumulation of pS129 in A53TxUb2-KO mice compared to A53T controls (Fig. 5D). We were also able to detect endogenous levels of murine pS129 in Ub2-hi and Ub2-KO mice despite the generally low amount of pS129 in non-diseased brains^29,33^ (Fig. 5E,F). We observed a trend towards decreased murine endogenous pS129 in Ub2-hi mice (p = 0.2; Fig. 5E,F) and no change in pS129 levels in Ub2-KO mice (p = 0.8; Fig. 5E,F). The ratio of endogenous pS129 to total endogenous Syn (Fig. 4D) revealed no significant differences between Non-Tg and Ub2-hi mice (p = 0.2; Fig. 5G) or Ub2-KO (p = 1; Fig. 5G). Together these results suggest that UBQLN2 regulates pS129 in vivo, both under normal conditions and in disease states.

## UBQLN2 targets pS129 α-syn to the proteasome for degradation

**Figure 6:**
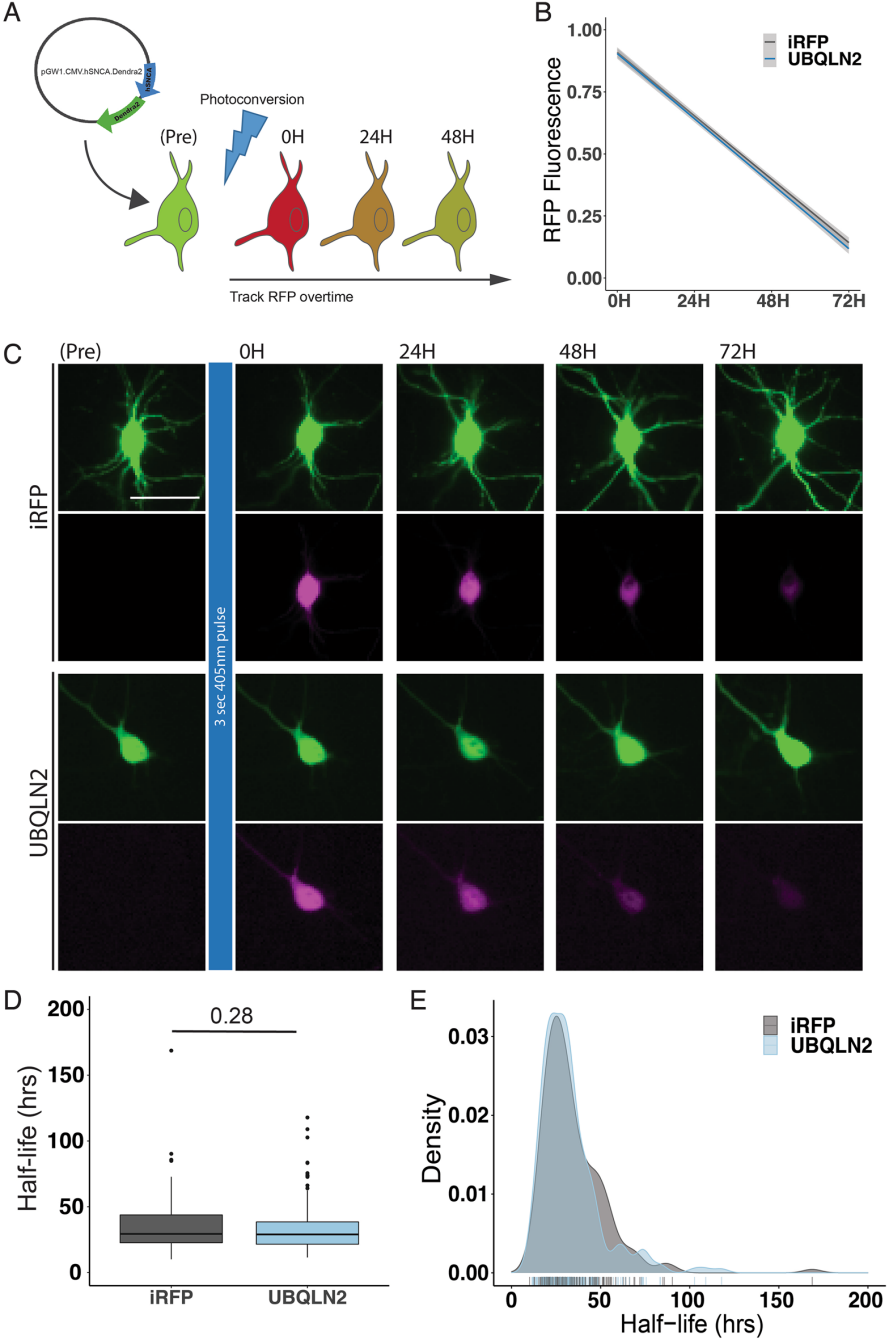
Optical Pulse Labeling reveals no effect of UBQLN2 on α-syn clearance in neurons. (**A**) Schematic of Optical Pulse labeling experiment. Rat primary cortical neurons were transfected with hSyn-Dendra2 and UBQLN2-iRFP or iRFP. Dendra2 was photoconverted 24 h post-transfection with 405 nm light, imaged immediately and every 24 h thereafter for 3 days. Scale bar 25 μm. (**B**) Optical pulse labeling reveals no change in red fluorescence intensity over time in neurons transfected with UBQLN2 (n = 213) compared to control (n = 182; p = 0.6). (**C**) Representative images of optical pulse labeling of hSyn-Dendra2 in rat primary cortical neurons transfected with hSyn-Dendra2 and UBQLN2-iRFP or iRFP. (**D**) Optical pulse labeling in primary rate neurons reveals no change in half-life of hSyn-Dendra2 upon transfection with UBQLN2 vs control (iRFP; p = 0.28). hSyn-Dendra2 half-life was determined by measuring the loss of photoconverted hSyn-Dendra2 (RFP) signal over time for each cell. (**E**) Density plot of hSyn-Dendra2 half-life measurements from individual neurons transfected with control (n = 182) or UBQLN2 (n = 213). Values were pooled from eight wells per condition, from each of two biological replicates and analyzed by the Kruskal–Wallis test.

To test whether overexpression of UBQLN2 leads to an increased rate of α-syn degradation we used optical pulse labeling to measure alpha-synuclein degradation over time^34^. For this experiment we used human α-syn tagged with Dendra2 (hSyn-Dendra2), a photoconvertible protein whose fluorescence changes from green to red following 405 nm light exposure (Fig. 6a). Rat primary cortical neurons were transfected with hSyn-Dendra2 and either UBQLN2-iRFP or iRFP. Following photoconversion, red fluorescence was tracked over time, allowing us to determine the rate of hSyn-Dendra2 turnover on a single-cell level. UBQLN2 overexpression had no effect on hSyn-Dendra2 decay compared to the iRFP control, suggesting that UBQLN2 does not play a significant role in nonpathogenic α-syn degradation (p = 0.6; Fig. 6b,c). Analysis of photoconverted hSyn-Dendra2 half-life further confirmed that UBQLN2 overexpression did not significantly affect normal α-syn degradation (p = 0.28; Fig. 6d,e). A limitation of optical pulse labeling, as applied here, is that we are unable to assess phosphorylated (pS129) α-syn at the same time.

**Figure 7:**
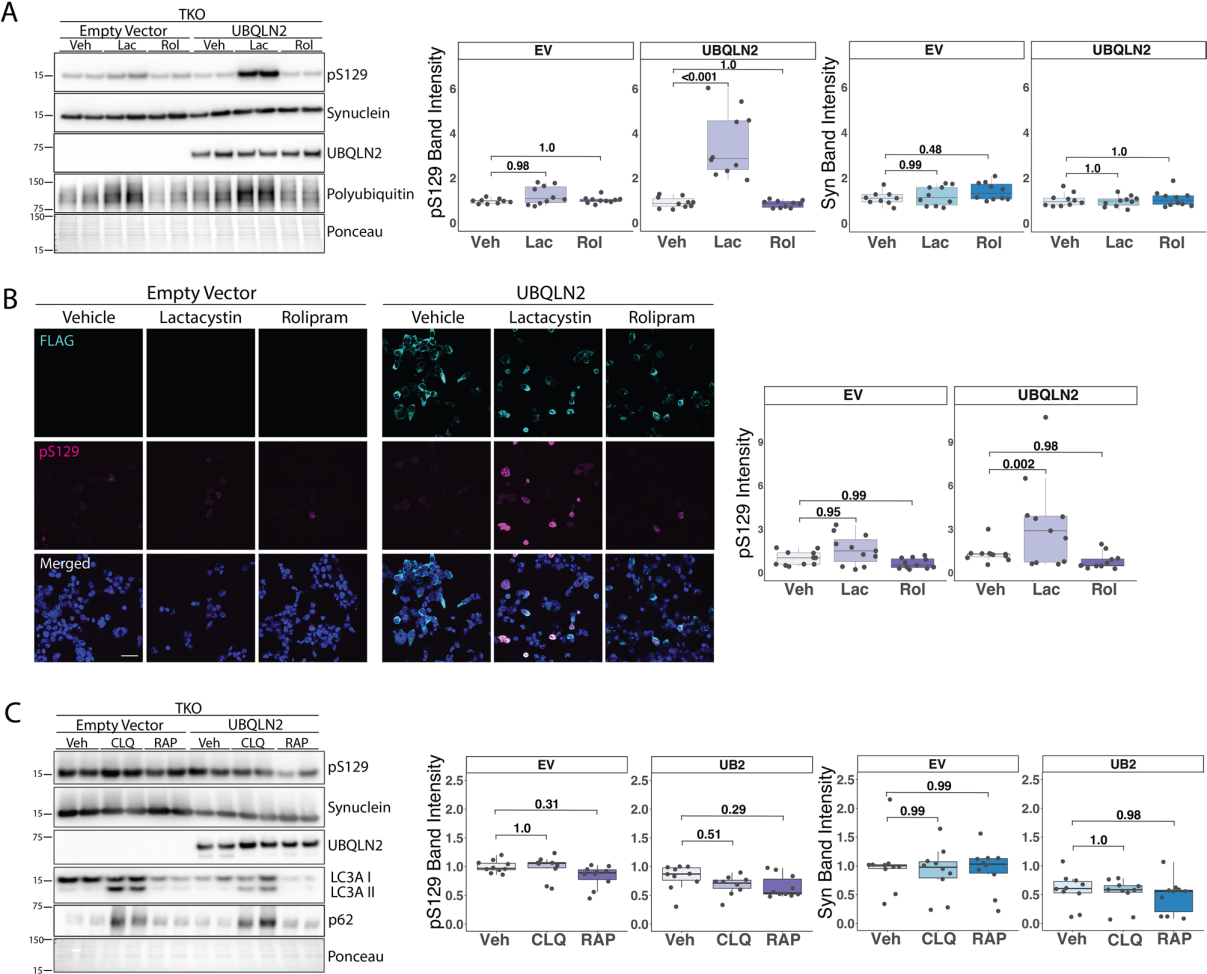
UBQLN2 targets pS129 α-syn to the proteasome for degradation. (**A**) Representative western blot of TKO cells overexpressing α-syn and transfected with either EV or UBQLN2 and treated with 10 μM lactacystin (Lac), 50 μM rolipram (Rol), or vehicle (Veh). Quantification of total α-syn and pS129 levels shows that pS129 accumulates in cells transfected with UBQLN2 and treated with Lac while total α-syn levels were not changed between groups (n = 9–10). Total α-syn and pS129 levels were each normalized to α-syn levels of EV, vehicle-treated cells. Western blots were cropped to focus on protein(s) of interest. (**B**) Representative images showing pS129 fluorescence in cells transfected with either EV or UBQLN2 and treated with either Veh, Lac, or Rol. Quantification of pS129 fluorescence intensity further supports that pS129 α-syn is increased when treated with Lac and in the presence of UBQLN2 (n = 11). pS129 fluorescence intensity was normalized to that of vehicle treated, EV cells. Scale bar 50 μm. (**C**) Representative western blot of TKO cells overexpressing α-syn and transfected with either EV or UBQLN2 and treated with 10 μM chloroquine (CLQ), 300 nM rapamycin (RAP), or vehicle (Veh). Quantification of pS129 or total α-syn levels reveals no differences between treatment groups (n = 10). Total α-syn and pS129 levels were each normalized to α-syn levels of EV-transfected, vehicle-treated cells. Uncropped Western blots are shown in Supplemental Figure S5.

α-syn and pS129 are regulated by both proteasomal and lysosomal degradation^35–37^. Though UBQLN2 may function in lysosomal protein degradation^16,18^, it is thought primarily to facilitate proteasomal degradation^16,22^. To investigate whether UBQLN2 targets α-syn to the proteasome for degradation, we treated TKO cells transfected to express α-syn and UBQLN2 or EV with either the proteasome inhibitor lactacystin^38^ (10 μM) or the proteasome activator Rolipram^39^ (50 μM). In the presence of overexpressed UBQLN2, proteasome inhibition led to a robust increase in pS129 (p ≤ 0.001) but not total α-syn (p = 1.0), whereas in the absence of UBQLN2 there was no such effect on pS129 (p = 0.98) or α-syn (p = 1.0) levels (Fig. 7A). Immunofluorescence supported the finding that overexpression of UBQLN2 leads to an accumulation of pS129 when the proteasome is inhibited (p = 0.002; Fig. 7B). These results suggest that while α-syn likely can be cleared by multiple, redundant quality control pathways, overexpressed UBQLN2 preferentially targets pS129 to the proteasome for degradation.

To determine whether UBQLN2 also can regulate α-syn or pS129 via lysosomal degradation, we treated TKO cells transfected to express α-syn and UBQLN2 or EV with either the autophagy inhibitor chloroquine^40^ (CLQ; 10 μM) or the autophagy activator rapamycin^41^ (RAP; 300 nM). pS129 levels were not significantly altered in cells transfected with EV and treated with either CLQ or RAP, compared to vehicle controls (Fig. 7C). Similarly, overexpression of UBQLN2 and subsequent treatment with CLQ or RAP had no effect on levels of pS129 levels (Fig. 7C). Our results suggesting that total α-syn is likely handled by multiple quality control pathways (Fig. 7A) is supported by our finding that inhibition or activation of lysosomal degradation in the presence or absence of UBQLN2 has no effect on total α-syn levels (Fig. 7C). These data suggest that UBQLN2 regulates α-syn via the proteasome when autophagy is impaired and highlight UBQLN2’s preferential regulation of pS129 via the proteasome.

## Discussion

Although UBQLN2 has been linked to several neurodegenerative diseases^10,15,31^ and is known to accumulate in Lewy bodies in disease^10^, whether this ubiquitin-linked quality control protein regulates α-syn under normal conditions or in disease states is unknown. Here we used human disease tissue, transgenic mouse models and cellular models to query whether UBQLN2 regulates α-syn. Our findings support a role for UBQLN2 in regulating levels of a key pathological form of α-syn, namely pS129.

UBQLN2 is intrinsically prone to form condensates and aggregates^10,15,21,25^ and accumulates in various neurodegenerative proteinopathies, including Lewy bodies in PD^10^. Our results reveal that UBQLN2 is more insoluble in PD. This is consistent with the previous finding that UBQLN2 colocalizes with aggregates of α-syn in PD^10^. Protein solubility may correlate with functionality, with decreased solubility correlating with decreased normal function. As UBQLN2 is sequestered in protein aggregates in disease, it may lose its normal function of regulating protein turnover. Conversely, as UBQLN2 solubility decreases, it could also undergo a gain-of-toxic-function^42^. In PD, UBQLN2 may be sequestered in aggregates, precipitating a loss of normal UBQLN2 function and in turn accelerating pS129 accumulation and potentially α-syn toxicity and disease progression.

Of the three brain-expressed UBQLNs, UBQLN2 has been most widely implicated in neurodegenerative diseases^15,31,43^ and our results supporting a significant role for UBQLN2, but not UBQLN1, in regulating α-syn are consistent with this concept. The UBQLNs are highly similar proteins except for the PXX domain that is unique to UBQLN2. Among UBQLNs, UBQLN2 and UBQLN1 are most similar in structure, sharing 79% homology^44^, but do not appear to share the role of regulating specific disease proteins such as α-syn and tau^45^. The PXX domain may be a driver of UBQLN2’s potential function as a regulator of such disease proteins. Of their many functions, proline-rich domains aid in protein binding^46^ so conceivably the PXX domain of UBQLN2 favors its interaction with client proteins independent of ubiquitination. Intriguingly, the PXX domain is also the site of most of the pathogenic UBQLN2 mutations that directly cause hereditary neurodegeneration^15^. Further investigations into the role of the PXX and other UBQLN2 domains are warranted to better understand how UBQLN2 shuttles pS129 to the proteasome for degradation.

A recent study assessing the effects of UBQLN2 on another disease-linked protein, tau, reported a greater effect of UBQLN2 on phosphorylated tau than on total tau^45^, reminiscent of our finding of a greater effect of UBQLN2 on pS129 α-syn than on total α-syn. Together, these studies support the view that UBQLN2 preferentially affects pathological forms of proteins, such as those that are phosphorylated or aggregated. Limitations of the current study include that fact that we have only assessed the most commonly identified site of pathological phosphorylation on α-syn, and the lack of phosphatase inhibitors in our biochemical analyses which could result in underrepresentation of the extent of UBQLN2’s effect on phosphorylated α-syn. Further mass spectrometry studies to evaluate additional phosphorylation sites^47–51^ (Ser87, Tyr136, Tyr125, etc.) will be needed to define a potentially broader role for UBQLN2 in regulating pathogenic forms of α-syn. Conceivably, UBQLN2 regulates protein phosphorylation, but there is no evidence that UBQLN2 regulates kinase or phosphatase activity^45^. Another possibility is that UBQLN2 preferentially targets aggregated proteins for degradation and disease aggregates are often enriched in phosphorylated proteins. Assessing this possibility in the current studies was complicated by the mild phenotype seen in hemizygous A53T mice and the lack of significant pathology until after 22 months of age^27^. An effect of UBQLN2 on A53T might be captured with the more robust homozygous mouse model or in vitro studies. Alternatively, the A53T mutation may alter UBQLN2’s regulation of α-syn considering that A53T α-syn is known to alter proteasome activity^52^, further complicating UBQLN2’s role on α-syn in PD.

UBQLN2 has been implicated in proteasomal and lysosomal degradation pathways^16,18,53^, pathways by which α-syn is regulated^6,9,36,54,55^. We sought to determine whether UBQLN2 acts on α-syn at the proteasomal or lysosomal level. In the absence of UBQLN2, pS129 appears to be cleared by other pathways when the proteasome is inhibited, but the presence of UBQLN2 restricts pS129 clearance to the proteasomal pathway. UBQLN2 may sequester pS129 at or near the proteasome, limiting accessibility to other clearance pathways. Our results further suggest that normal (nonpathogenic) α-syn can be regulated by redundant protein quality control pathways and that the presence or absence of UBQLN2 does not greatly affect normal α-syn degradation by those pathways. This may reflect a limited role for UBQLN2 in normal α-syn degradation or an ability of UBQLN2 to regulate normal α-syn via multiple clearance pathways. The former is supported by our kinetic studies showing that UBQLN2 does not significantly alter normal α-syn half-life. Further optical pulse labeling kinetic studies employing a phospho-mimetic of pS129 could help determine the basis of UBQLN2’s selective effect on this pathogenic form of α-syn.

In summary, we have established a role for UBQLN2 in regulating a pathological form of α-syn by targeting it to proteasomal degradation. This knowledge of the role of UBQLN2 in regulating pS129 sheds light on one potential reason why UBQLN2 is found in Lewy bodies in synucleinopathies: to decrease pS129 levels. Although further studies will be needed to understand the differential roles of UBQLN2 in regulating normal, post-translationally modified, and mutant forms of α-syn, our results here defining UBQLN2 as a regulator of pS129 α-synuclein point to UBQLN2 as a potentially important modifier of synucleinopathies and as a component of altered proteostasis in neurodegenerative disease.

## Methods

### Plasmids and siRNAs

The pCMV4-FLAG-UBQLN2 plasmid (p4455 FLAG-hPLIC-2; Addgene plasmid # 8661) and pCS2-FLAG-UBQLN1 plasmid (p4458 FLAG-hPLIC-1; Addgene plasmid # 8663) were gifts from Peter Howley^56^. The pAAV-MCS-α-synuclein plasmid (pAAV asyn WT; Addgene plasmid # 36055) was a gift from Hilal Lashuel^48^. Control empty vector plasmid for cell transfection experiments was pCMV-HA was used as a control for UBQLN2 clearance experiments. For UBQLN2 and UBQLN1 knockdown experiments, Dharmacon SMARTpool siGENOME siRNA against UBQLN1 and UBQLN2 and MISSION siRNA Universal Negative Control (Sigma) were used. The pGW1-hSyn-Dendra2 plasmid was synthesized by VectorBuilder. Creation of the pGw1-UBQLN2-iRFP and pGW1-iRFP plasmids is described in Sharkey et al.^25^.

### Cell culture

Human embryonic kidney 293 (HEK-293) cells were cultured in high glucose DMEM (Gibco), supplemented with 10% FBS, 10 mM glutamine, and 100 U/ml penicillin/streptomycin. UBQLN1/2/4 triple knockout (TKO)^30^, UB1 rescue^30^, UB2 rescue, and TREX HEK-293 (TREX) cell lines were cultured in high glucose DMEM, supplemented with 10% FBS, 10 μg/ml blasticidin, and 100 μg/ml hygromycin.

### Cell transfection

Cells were transfected with DNA plasmids using Lipofectamine-2000 according to the manufacturer’s instructions. Cells used for downstream Western blot analyses were lysed in RIPA buffer (Thermo Scientific) with protease inhibitor cocktail (catalog no. 11873580001; Sigma Aldrich). Cells used for immunofluorescence analyses were fixed in chilled methanol for 20 min at – 20 °C prior to staining. For UBQLN rescue experiments, TREX, TKO, UB1 rescue, and UB2 rescue cell lines were treated with 10 ng/ml doxycycline to induce UBQLN 1 or 2 expression in the respective cell lines 24 h prior to transfection.

### Human disease brain tissue

Frozen brain tissue from the cingulate cortex was obtained from subjects with PD, DLB and age-matched control subjects (Table 1) from the Michigan Brain Bank (University of Michigan, Ann Arbor, MI, USA). Brain tissue was collected following a standard protocol in which patients gave informed consent for autopsy and, upon death, a Michigan Brain Bank physician confirmed this existing consent with the next of kin before proceeding.

**Table 1:** Synucleinopathy samples used for analysis of UBQLNs.

Synucleinopathy tissue			
Diagnosis	Sex	Age	PMI (h)
Control	Female	83	21
Control	Female	80	19
Control	Male	100	3
Control	Female	96	18
Control	Male	75	9
Control	Male	65	24
Control	Female	83	Not recorded
Control	Male	71	4
Control	Female	80	5
PD	Male	78	22
PD	Male	74	14
PD	Female	71	7
PD	Male	86	10
PD	Female	74	6
PDD	Male	81	16
DLB	Male	78	12
DLB	Female	82	10
DLB	Male	84	5
DLB	Female	68	24
DLB	Male	66	8
DLB	Male	86	Not recorded
DLB	Male	66	15
DLB	Male	71	5
DLB	Female	80	4
DLB	Female	57	9
DLB	Female	84	6
DLB	Male	87	13
DLB	Male	72	18
DLB	Female	71	12

Protocols were approved by the Institutional Review Board of the University of Michigan and abide by the Declaration of Helsinki principles. Samples were examined at autopsy by neuropathologists for diagnosis.

### Mouse models

This study was conducted in a facility approved by the American Association for the Accreditation of Laboratory Animal Care, and all experiments were performed in accordance with the National Institutes of Health *Guide for the Care and Use of Laboratory Animals* and approved by the Institutional Animal Care and Use Committee of the University of Michigan and reported in accordance with ARRIVE guidelines^57^. Mice were housed at the University of Michigan animal care facility and maintained according to U.S. Department of Agriculture standards (12 h light/dark cycle with food and water available ad libitum). Twelve-month-old hemizygous UBQLN2 transgenic mice generated in the laboratory^58^ and non-transgenic littermates were euthanized with isofluorane, perfused with PBS and brains were removed for analysis. Additionally, we acquired brain from 10-month-old homozygous A53T-mutated α-synuclein M83 mice (B6;C3-Tg-Prnp-SNCA-A53T 83Vle/J; The Jackson laboratory stock #004479)^27^, and non-transgenic littermates were a gift from Dr. Rakez Kayed. Both male and female mice were included in analyses.

Hemizygous UBQLN2-high^58^ and UBQLN2-KO^58^ mice were crossed with hemizygous A53T-mutated α-synuclein M83 mice (B6;C3-Tg-Prnp-SNCA-A53T 83Vle/J; The Jackson laboratory stock #004479)^27^. 12 months of age male and female, UBQLN2-high transgenic, UBQLN2 KO, A53T, A53TxUBQLN2-high, A53TxUBQLN2-KO, and non-transgenic littermate controls were included in analyses. Prior to analyses, mice were euthanized with isofluorane and perfused with PBS prior to removing the brain for analysis. Following dissection, brains were divided sagittally. One half was flash frozen on dry ice and stored at – 80 C for biochemical studies. The other half was fixed in 4% paraformaldehyde at 4 °C before undergoing a 10–30% sucrose gradient at 4 °C until saturated and then flash frozen in OCT and stored at − 80 °C until sectioning. 25 μM sagittal sections were taken from fixed hemispheres using a cryostat. Sections were stored at − 20 °C in cryoprotectant prior to immunofluorescence studies. Both male and female mice were included in analysis.

### Western blot analysis

Cells were lysed in RIPA buffer (ThermoScientific) with protease inhibitor cocktail then sonicated for 5 min in chilled water before being centrifuged at 10,000 rcf for 10 min at 4 °C. Cell lysates were loaded (without boiling) on precast NuPAGE 4–12% Bis–Tris gels (Invitrogen) for SDS-PAGE analysis. Gels were subsequently transferred onto nitrocellulose membranes and stained with ponceau then blocked for 1 h at room temperature with 10% nonfat dry milk in TBS-T buffer. Membranes were then probed overnight at 4 °C in anti-Ubiquilin-2 (Novus Biologicals; 1:2000), anti-FLAG, clone M2 (Sigma, 1:2000), anti-α-synuclein (Invitrogen; 1:50,000), anti-pS129-α-synuclein (Abcam; 1:3000), anti-FK1 (Enzo, 1:2000), or anti-GAPDH (Millipore; 1:5000) diluted in 5% nonfat dry milk. HRP-conjugated goat anti-rabbit IgG, goat anti-mouse IgG, or goats anti-mouse IgM (1:5000) secondary antibodies were used for detection as appropriate. EcoBright ECL (Innovative Solution) was used to visualize bands, which were normalized to corresponding ponceau smear. All quantification of immunoblots were performed by densitometric analysis using ImageJ software (National Institutes of Health). Analyses were completed in triplicate and analyzed by one-way ANOVA with the Tukey post hoc test for multiple comparisons. Representative western blots were cropped for ease of visualization with full un-cropped western blot images included in supplemental figures.

Mouse and human brain samples were homogenized in PBS with protease inhibitor cocktail (catalog no. 11873580001; Sigma Aldrich), using a 1:3 dilution of tissue: PBS (w/v). 100 μL of homogenate was removed and lysed using RIPA buffer. The remaining lysate was centrifuged at 10,000 rcf for 10 min at 4 °C. Supernatants (PBS-soluble fraction) were collected. Pellets were resuspended in PBS with protease inhibitor cocktail, centrifuged at 10,000 rcf for 10 min at 4 °C and supernatants were added to the PBS-soluble fractions then aliquoted, snap-frozen, and stored at − 80 °C until use. Remaining pellet was resuspended in 1% sarkosyl in PBS with protease inhibitor, vortexed for 1 min, and incubated at room temperature for 1 h then stored at − 80 °C overnight. The next day, samples were thawed then water sonicated for 5 min and centrifuged for 30 min at 14,000 rpm at 4 °C. Supernatants were collected as insoluble fraction. Brain extracts containing 15 μg of total protein were boiled at 98 °C for 1 min then loaded on precast NuPAGE 4–12% Bis–Tris gels (Invitrogen) for SDS-PAGE analysis. Gels were subsequently transferred onto nitrocellulose membranes and blocked for 1 h at room temperature with 10% nonfat dry milk in TBS-T buffer. Membranes were then probed overnight at 4 °C in anti-Ubiquilin-2 (Novus Biologicals; 1:2000), anti-FLAG, clone M2 (Sigma, 1:2000), anti-α-synuclein (Invitrogen; 1:50,000), anti-pS129-α-synuclein (Abcam; 1:5000), anti-FK1 (Enzo, 1:1000) diluted in 5% nonfat dry milk. HRP-conjugated goat anti-rabbit IgG, goat anti-mouse IgG, or goat anti-mouse IgM (1:5000) were used for detection as appropriate. EcoBright ECL (Innovative Solutions) was used to visualize bands, which were normalized to corresponding ponceau smear. All quantification of immunoblots was performed by densitometric analysis using ImageJ software (National Institutes of Health). Analyses were completed in triplicate and analyzed by one-way ANOVA with the Tukey post hoc test for multiple comparisons.

### Immunofluorescence

Cells were fixed in chilled methanol for 20 min at − 20 °C then washed in 1× PBS. Cells were then permeabilized with 0.5% triton-X 100 then blocked in 5% goat serum for 1 h prior to incubation in either anti-UBQLN2 (Novus Biologicals; 1:200), anti-UBQLN1 (Novus Biologicals, 1:100), or anti-FLAG (Sigma; 1:300) and anti-α-synuclein (BD Biosciences, 1:250) overnight at 4 °C. The following day, cells were washed three times in 1× PBS for 10 min then incubated in goat anti-rabbit IgG Alexa Fluor 568 (Invitrogen; 1:500) and goat anti-mouse IgG Alexa Fluor 488 (Invitrogen; 1:500) for 1 h. Cells were then washed three times in 1× PBS for 10 min and incubated in DAPI (Sigma) for 5 min at room temperature. Cells were washed in 1× PBS for 5 min three times then mounted with Prolong Gold Antifade Reagent (Invitrogen). Slides were images using a IX71 Olympus inverted microscope and analyzed for α-syn fluorescence using the cell counter toll in ImageJ.

Brain Sections were washed in 1× PBS at 4 °C overnight to wash out storage cryoprotectant. The next day, sections were subjected to heated antigen retrieval in 10 mM Citrate Buffer (pH 6) at 80 °C for 20 min then allowed to cool to room temperature for 15 min. Sections were then washed in 1× PBS-T three times for 5 min each followed by permeabilization in 0.5% triton-X 100. Slices were then incubated in M.O.M. Mouse IgG Blocking Reagent (Vector Laboratories) for 2 h and then incubated in either anti-a-synuclein (Invitrogen, 1:5000), UBQLN2 (Novus; 1:250) overnight at 4 °C. The next day, sections were washed three times in 1× PBS-T and then incubated in secondary antibody then washed three time in 1× PBS-T. Section were then incubated in DAPI for 15 min followed by three more washes in 1× PBS-T. Once on slides, sections were cover slipped using ProLong Gold Antifade Reagent. Images were taken using a Nikon A1 inverted confocal microscope and analyzed for total fluorescence using ImageJ.

### Immunohistochemistry

Brain sections were washed with 1× PBS at 4 °C overnight to wash out storage cryoprotectant. The next day, sections were subjected to heated antigen retrieval in 10 mM Citrate Buffer (pH 6) at 80 °C for 15 min then allowed to cool to room temperature for 10 min. We then incubated cells in 1% hydrogen peroxide for 30 min to quench endogenous peroxidase activity. Sections were washed in 1× PBS for 10 min followed by 15 min in 0.1% Triton X-100 and then washed for 15 min in 0.5% bovine serum albumin. Sections were blocked for 1 h in 5% normal goat serum then washed in 1× PBS for 10 min followed by a 15 min wash in 0.1% Triton X-100. Sections were incubated in anti-pS129 primary antibody (Abcam, 1:1000) diluted in 0.5% BSA overnight at 4 °C. The next day, sections was washed in 0.1% Triton X-100 for 15 min followed by a 15 min was in 0.5% BSA and then incubated in secondary antibody for 1.5 h. Sections were then washed for 15 min in 0.1% Triton X-100 followed by a 15-min wash in 0.5% BSA. Sections were then incubated in ABC buffer (Elite ABC-HRP kit, Vector labs) in the dark for 1 h. Sections were then washed 3 × 5 min in 1× PBS. Sections then underwent a DAB reaction using ImmPACT DAB as per manufacturer’s instructions (Vector Labs) followed by 3 × 10 min washes in 1× PBS. Sections were then mounted on Superfrost Plus slides with 1 × PBS and air dried overnight. The next day, sections were rinsed in ultra-pure water (MilliQ) and then dehydrated in 70% ethanol for 1 min, 95% ethanol for 2 min, and 100% ethanol for 2 x 3 min, followed by 3 × 5-min incubations in xylenes. Coverslips were then placed on sections with DPX mounting media. Once dried, slides were imaged using an Olympus BX51 microscope.

### Optical pulse labeling

Cortical primary neurons were dissected from rat pups at embryonic day 20. Neurons were plated on maninin/poly-d-lysine coated 96 well plates^59–62^ at a density of 1 × 10^5^ cells/well in NEUMO photostable media supplemented with SOS (Cell Guidance Systems). Neurons were transfected with hSyn-Dendra2 and UBQLN2-iRFP or iRFP plasmids using lipofectamine 2000 as previously described^59–62^.

Imaging of primary neurons was accomplished using a Nikon Eclipse Ti inverted microscope equipped with Semrock filter sets, Sutter Lambda 421 multi-LED light source, and an Andor Zyla 4.2(+) sCMOS camera (Oxford Instruments), enclosed in a custom-built environmental chamber to maintain a temperature of 37 °C and 5% CO_2_ levels. All stage movements, filter switching, illumination and image acquisition were controlled through μManager with original code written in BeanShell, as described previously. Image processing and analyses were accomplished through dedicated software written expressly for these purposes^63^. Fluorescence was initially measured 24 h following transfection. We then used a 3 s pulse of 405 nm light for Dendra2 photoconversion and obtained images again 3 h following photoconversion and then every 24 h for the following 3 days as previously described^34,59,60^. Only cells that survived the entire duration of the experiment were included for further analysis. The half-life of hSyn-Dendra2 in individual neurons was determined by fitting the time-dependent decline in photoconverted (red) hSyn-Dendra2 fluorescence for each cell to a first-order exponential decay curve.

### Experimental design and statistical analysis

The accepted level of significance for all analyses was p ≤ 0.05. All analyses comparing two groups were analyzed using student’s t-test and comparisons between three or more groups were completed by one-way ANOVA and the Tukey post-hoc test for multiple comparisons or, when applicable, the non-parametric Kruskal Wallis test. Data are expressed as means ± SEM. p values for overall statistical analysis are displayed in analyses that did not show a significant difference and individual post-hoc comparison p values are displayed for significant analyses. Both male and female mice were included in our analyses and, as no sex differences were observed, male and female data points were grouped together for statistical analyses. Data were analyzed using RStudio.

## Supplementary Information

Supplementary Information.

## Data Availability

The datasets used during the current study are available from the corresponding author on reasonable request.
