# Supplementary material for: Ubiquilin-2 regulates pathological alpha-synuclein
Source: Sci Rep. 2023 Jan 6;13:293. doi: 10.1038/s41598-022-26899-0 (PMC9823102; doi:10.1038/s41598-022-26899-0)
Supplement: Supplementary file 1 — Supplementary Information. [file 41598_2022_26899_MOESM1_ESM.pdf]

Supplemental Information

Title: UBQLN2 regulates pathological alpha-synuclein

Authors: Stephanie S. Sandoval-Pistorius, Julia E. Gerson, Jaimie H. Ryou, Nyjerus Liggans, Kulin Oak, Xingli Li, Keyshla Y. Negron-Rios, Svetlana Fischer, Henry Barsh, Emily V. Crowley, Mary E. Skinner, Lisa M. Sharkey, Sami J. Barmada, and Henry L. Paulson.

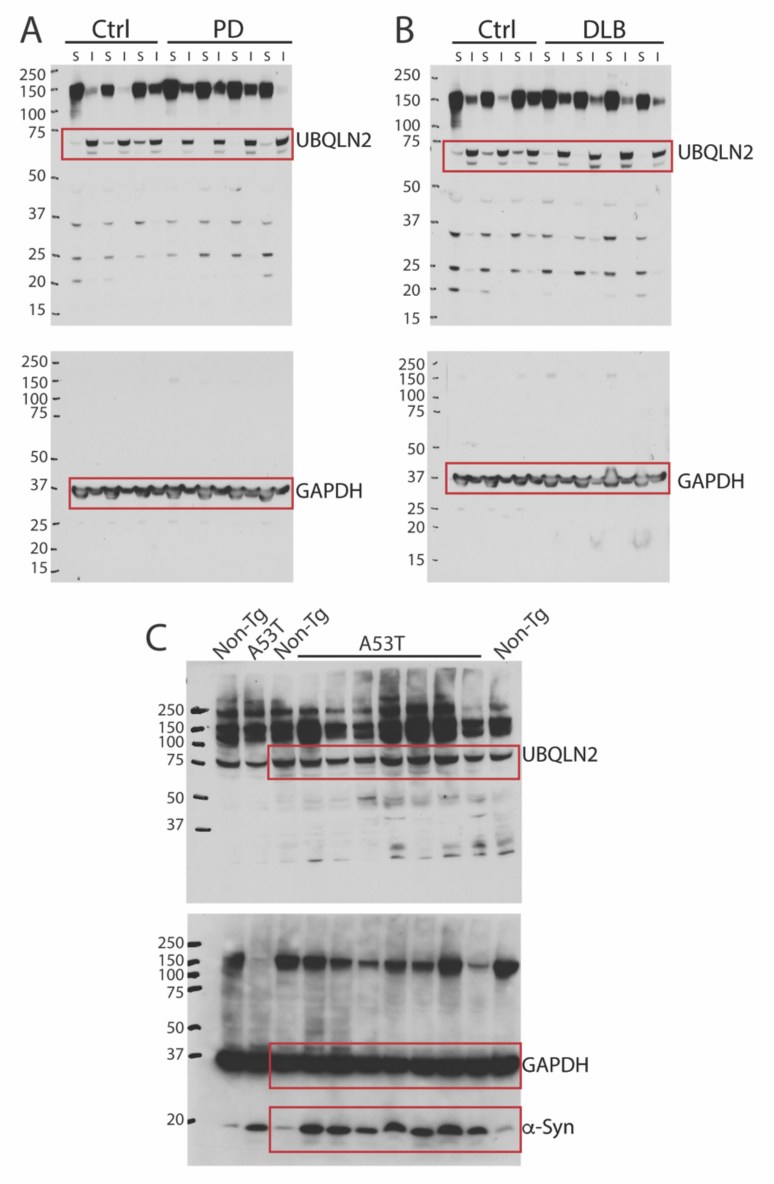

Supplementary Figure S1

Full, uncropped Western blots from Figure 1. Red boxes indicate where images were cropped in the main figure.

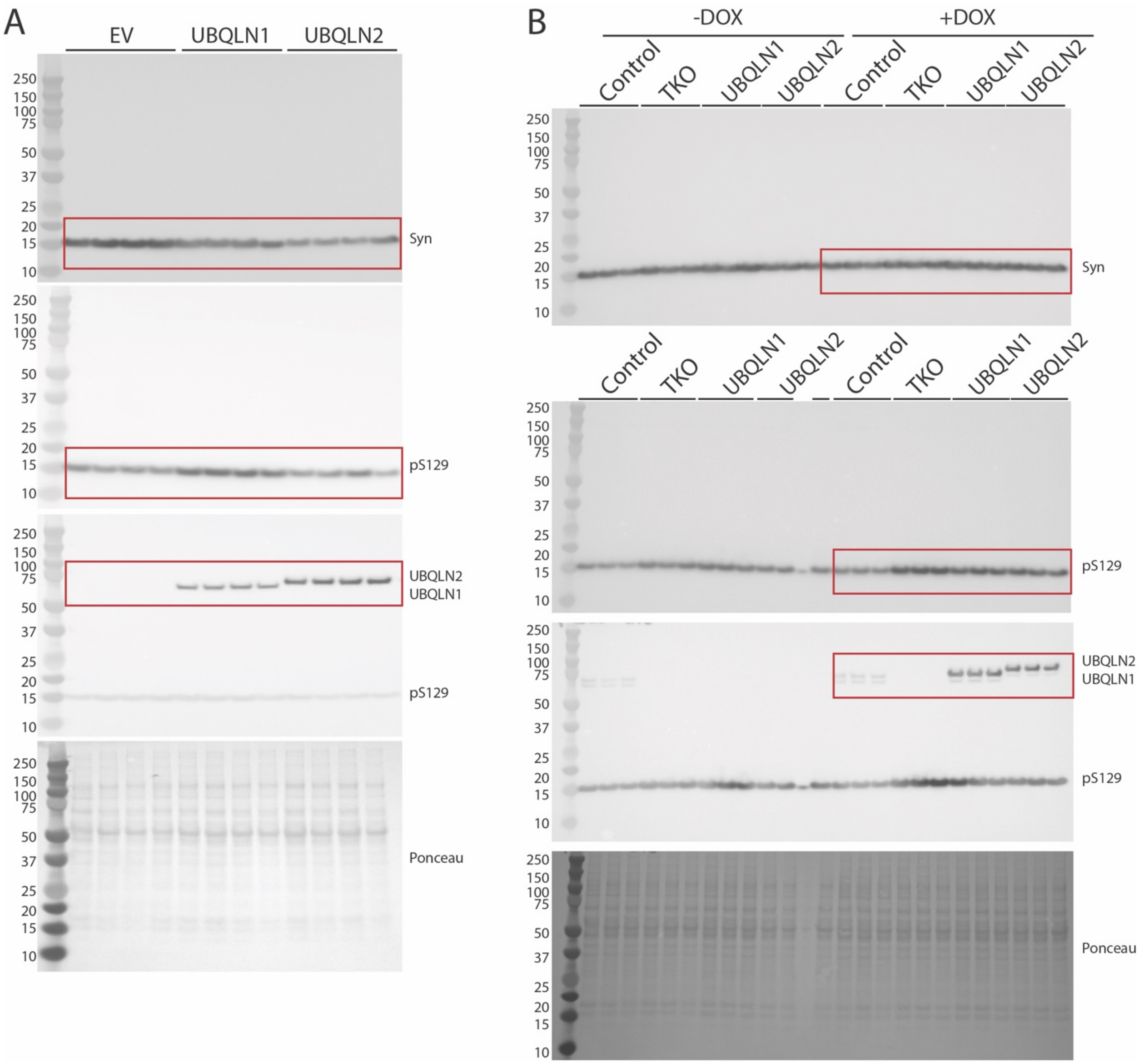

Supplemental Figure S2.

Full uncropped Western blots from Figure 2. Red boxes indicate where images were cropped in the main figure.

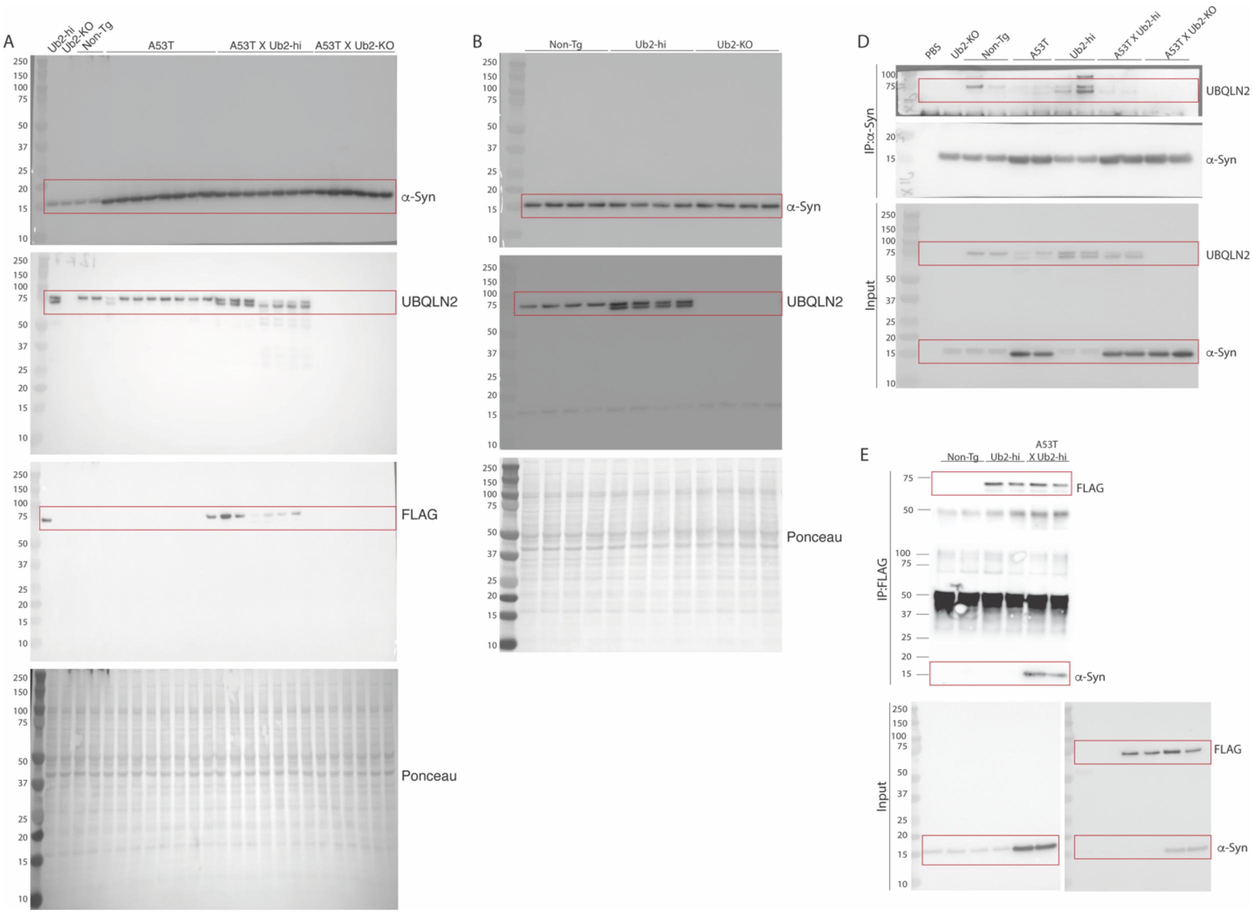

Supplementary Figure S3.

Full, uncropped Western blots from Figure 4. Red boxes indicate where images were cropped in the main figure.

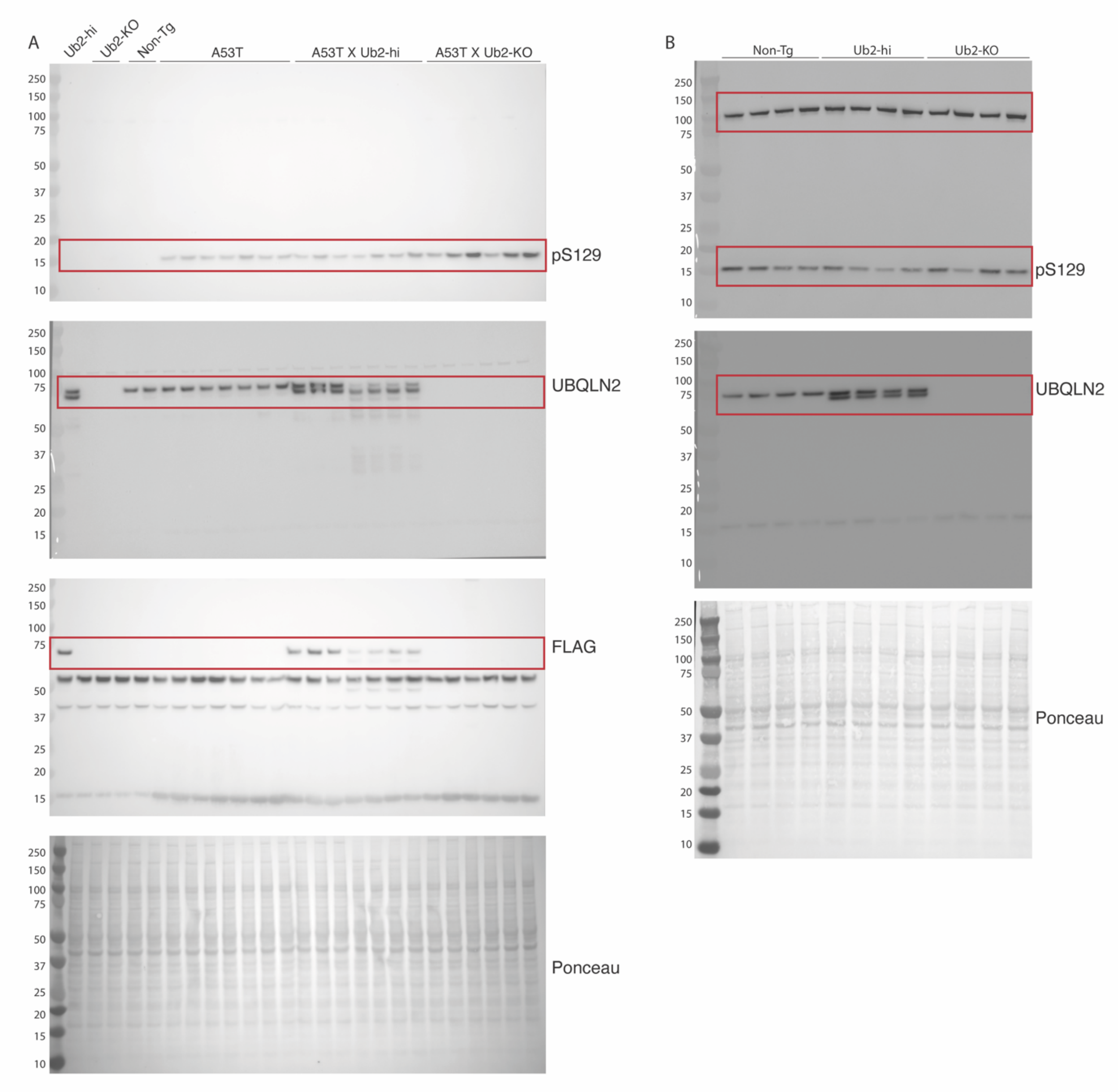

Supplementary Figure S4.

Full, uncropped Western blots from Figure 5. Red boxes indicate where images were cropped in the main figure.

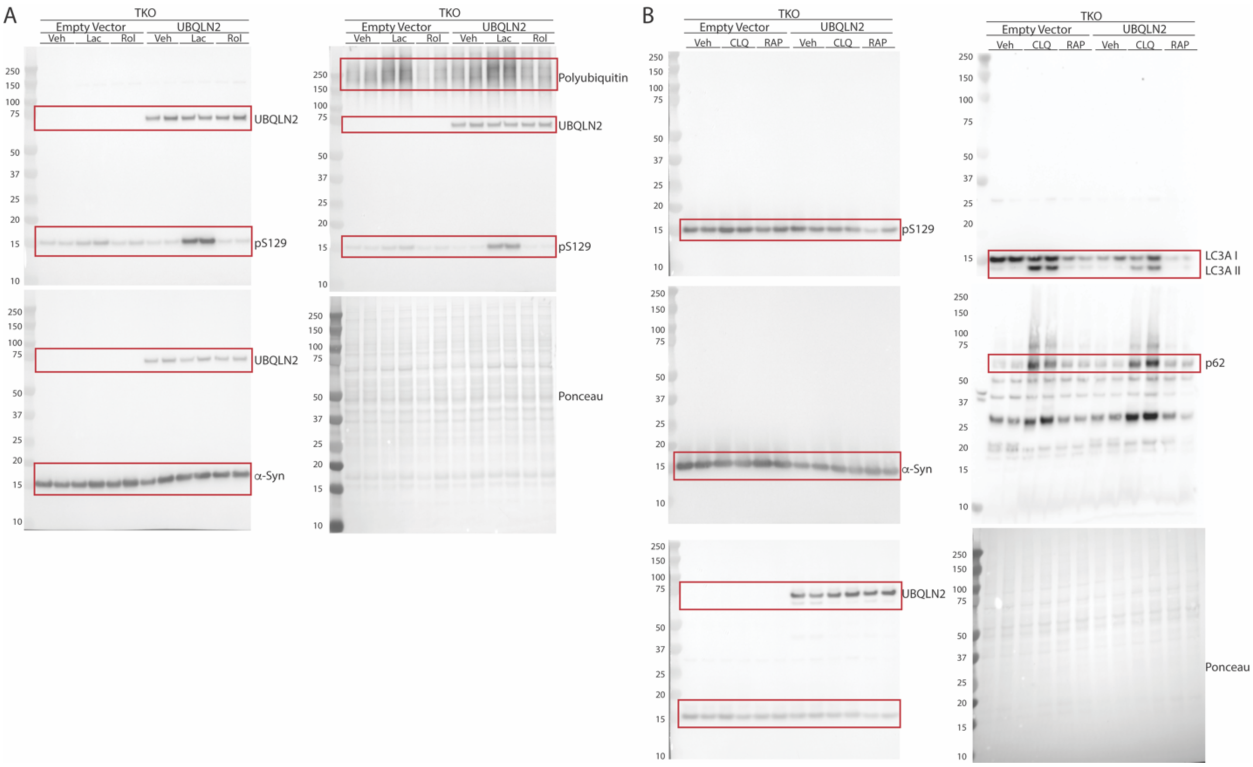

Supplementary Figure S5.

Full, uncropped Western blots from Figure 7. Red boxes indicate where images were cropped in the main figure.
